# ^18^F-FDG-PET/CT in Patients with Advanced, Radioiodine Refractory Thyroid Cancer Treated with Lenvatinib

**DOI:** 10.3390/cancers13020317

**Published:** 2021-01-16

**Authors:** Freba Ahmaddy, Caroline Burgard, Leonie Beyer, Viktoria Florentine Koehler, Peter Bartenstein, Matthias P. Fabritius, Thomas Geyer, Vera Wenter, Harun Ilhan, Christine Spitzweg, Andrei Todica

**Affiliations:** 1 Department of Nuclear Medicine, University Hospital, LMU Munich, 81377 Munich, Germany; Freba.Ahmaddy@med.uni-muenchen.de (F.A.); Caroline.Burgard@med.uni-muenchen.de (C.B.); Leonie.Beyer@med.uni-muenchen.de (L.B.); Peter.Bartenstein@med.uni-muenchen.de (P.B.); Vera.Wenter@med.uni-muenchen.de (V.W.); Harun.Ilhan@med.uni-muenchen.de (H.I.); 2 Department of Internal Medicine IV, University Hospital, LMU Munich, 81377 Munich, Germany; Viktoria.Koehler@med.uni-muenchen.de (V.F.K.); Christine.Spitzweg@med.uni-muenchen.de (C.S.); 3 Comprehensive Cancer Center (CCC LMU), University Hospital, LMU Munich, 81377 Munich, Germany; 4 Interdisciplinary Center for Thyroid Carcinoma (ISKUM), University Hospital, LMU Munich, 81377 Munich, Germany; 5 Department of Radiology, University Hospital, LMU Munich, 81377 Munich, Germany; Matthias.Fabritius@med.uni-muenchen.de (M.P.F.); Thomas.Geyer@med.uni-muenchen.de (T.G.)

**Keywords:** differentiated thyroid cancer, radioiodine refractory, Lenvatinib, ^18^F-FDG-PET/CT

## Abstract

**Simple Summary:**

In patients with advanced radioiodine refractory differentiated thyroid carcinoma (DTC), therapeutic options are limited. In the “Study of (E7080) Lenvatinib in Differentiated Cancer of the Thyroid (SELECT)”, Lenvatinib significantly prolonged the progression-free survival, resulting in a more frequent use in clinical practice for this patient group. Due to considerable side effects, an accurate assessment of response to treatment is crucial in these patients. Therefore, we aimed to improve treatment individualization and reduce unnecessary therapies by selecting patients who will most likely benefit from Lenvatinib treatment using 2-deoxy-2-[^18^F] fluoro-D-glucose positron-emission-tomography/computed-tomography.

**Abstract:**

Background: The tyrosine kinase inhibitor (TKI) Lenvatinib represents one of the most effective therapeutic options in patients with advanced radioiodine refractory differentiated thyroid carcinoma (DTC). We aimed to assess the role of 2-deoxy-2-[^18^F] fluoro-D-glucose positron-emission-tomography/computed-tomography (^18^F-FDG-PET/CT) in the monitoring of functional tumor response compared to morphological response. Methods: In 22 patients, a modified Positron Emission Tomography Response Criteria In Solid Tumors (mPERCIST) evaluation before treatment with Lenvatinib and at 3 and 6 month follow up was performed. Further PET-parameters and morphologic tumor response using Response Evaluation Criteria in Solid Tumors (RECIST) 1.1 were assessed and their prediction of progression-free survival (PFS) and disease-specific survival (DSS) was evaluated. Results: Most patients were rated stable in morphological evaluation and progressive using a metabolic response. All patients who responded to therapy through RECIST showed a decline in nearly all Positron Emission Tomography (PET)-parameters. For both time-points, non-responders according to mPERCIST showed significantly lower median PFS and DSS, whereas according to RECIST, only DSS was significantly lower. Conclusion: Tumor response assessment by ^18^F-FDG-PET outperforms morphological response assessment by CT in patients with advanced radioiodine refractory DTC treated with Lenvatinib, which seems to be correlated with clinical outcomes.

## 1. Introduction

Differentiated thyroid cancer (DTC) is the most frequent endocrine malignancy comprising the papillary (PTC), follicular (FTC) and poorly differentiated (PDTC) histological subtypes [1]. Although most patients with DTC can be cured by total thyroidectomy and radioiodine treatment (RAI; 10-year survival rate >90%) [2], approximately 5–10% of patients with DTC develop an aggressive disease with distant metastases and loss of 131-iodine avidity [3,4]. Inoperable metastatic and/or radioiodine refractory DTC is associated with a less favorable prognosis, with 10-year survival rates between 25% and 40%. Distant metastases most frequently occur in the lungs (50%), bones (25%) or both (20%) and less frequently at other sites (5%) [1,3,5,6]. In these patients, therapeutic options are limited. 

Tyrosine kinase inhibitors (TKIs), which inhibit VEGF (vascular endothelial growth factor) receptor signaling and tumor angiogenesis, improve progression-free survival (PFS) in patients with structurally progressive, radioiodine refractory DTC [7,8,9,10]. Lenvatinib is an oral, multitargeted TKI targeting VEGFRs 1–3, FGFRs (fibroblast growth factor receptors) 1–4, PDGFR (platelet-derived growth factor receptor) α, RET (rearranged during transfection receptor tyrosine kinase) and c-KIT (receptor for stem cell factor) signaling networks [11,12]. In the placebo-controlled phase 3 “Study of (E7080) Lenvatinib in Differentiated Cancer of the Thyroid (SELECT)”, Lenvatinib significantly prolonged progression-free survival (PFS), resulting in approval by the US Food and Drug Administration and European Medicines Agency in 2015 [13].

Accurate assessment of treatment response is crucial to discriminate between responders and non-responders thereby avoiding inefficient therapy and its potential adverse effects. The current standard for monitoring tumor response is measurement of change in tumor size based on anatomical imaging techniques such as computed tomography (CT); this technique has been most frequently assessed by the Response Evaluation Criteria in Solid Tumors (RECIST) [14], which were updated (RECIST 1.1 criteria) in 2009 [15]. As newer cancer treatments are cytostatic rather than cytotoxic, functional changes are expected to precede the morphologic changes and therefore 2-deoxy-2-[^18^F] fluoro-D-glucose positron emission tomography/computed tomography (^18^F-FDG-PET/CT) has the potential to improve diagnostic accuracy and prediction of the course of tumor development [16,17]. Wahl et al. described the Positron Emission Tomography Response Criteria In Solid Tumors (PERCIST) in 2009 to provide a structured guidance for response assessment using ^18^F-FDG-PET [18]. However, data on potential ^18^F-FDG-PET/CT applications in staging and restaging of advanced radioiodine refractory DTC is limited.

The aim of this study was to assess the role of ^18^F-FDG-PET in the monitoring of functional tumor response (modified PERCIST 1.0 and quantitative PET-parameters) in comparison to morphological response (RECIST 1.1) in combined ^18^F-FDG-PET/CT and thereby predicting both PFS and disease-specific survival (DSS) in patients with advanced radioiodine refractory DTC receiving Lenvatinib treatment.

## 2. Results

### 2.1. Patient Characteristics

Twenty-two patients (11 female) with advanced radioiodine refractory DTC fulfilled the inclusion criteria. The mean age at primary presentation was 53.1 ± 13.1 years and 60.8 ± 13.7 years at Lenvatinib treatment start. For most of the patients (*n* = 12), the histological subtype was FTC, followed by 7 PDTC and 3 PTC patients. Ten patients initially presented with stage IV, 9 patients with stage III and 3 patients with stage II DTC according to the seventh edition of the American Joint Committee on Cancer (AJCC) tumor-node-metastasis (TNM) staging system. A total of 4 out of 12 patients with FTC showed vascular invasion. Histopathological data of study patients are presented in Tables S1 and S3 in the supplements. Seven patients already showed distant metastases at primary presentation (5/7 patients with pulmonary metastases). At Lenvatinib treatment start, the majority of the patients (*n* = 20) showed advanced metastatic disease and in 14 patients, more than one site was affected. Metastatic sites were most frequently the lung (*n* = 19), followed by lymph nodes (*n* = 12), bone (*n* = 5), pleura (*n* = 2), liver (*n* = 2), subcutaneous metastases (n = 1), brain (*n* = 1) and kidney (*n* = 1). Local recurrence occurred in 6/22 patients (2/6 without distant metastases). Mean Thyroglobulin (Tg)-level at treatment start was 3950 ± 7062 ng/mL. 

Prior to start of Lenvatinib treatment, all patients had total thyroidectomy with or without lymphadenectomy. Surgery had been followed by RIT for remnant ablation. Additionally, 15/22 patients received more than two courses of RIT (range 2–11), 12/22 patients had undergone further surgery/metastasectomy, 13/22 had received external beam radiotherapy, 9/22 patients had been treated with TKIs other than Lenvatinib (Sorafenib *n* = 7, Pazopanib *n* = 3, Cabozantinib *n* = 2), 2/22 had received chemotherapy and one patient had undergone redifferentiation therapy with Dabrafenib. Single-patient characteristics are presented in Table S1 in the supplemental information.

In 14/22 patients, treatment was initiated at full dose (daily dose of 24 mg per day) and 8/22 patients received reduced dosage (20 mg per day *n* = 1, 18 mg per day *n* = 3, 14 mg per day *n* = 3, 10 mg per day *n* = 1). The mean Lenvatinib dose during treatment was 17.7 ± 4.2 mg/die. Dose interruptions and incremental reductions in the dose because of toxic effects were seen in 16/22 patients. The most frequent adverse effects were hypertension (*n* = 14), fatigue (*n* = 12), decreased appetite (*n* = 12), diarrhea (*n* = 11) and nausea (*n* = 6). 

### 2.2. Evaluation of Treatment Response 

The median duration of treatment with Lenvatinib was 11.0 months (interquartile range, IQR 25% 5.2–IQR 75% 26.3). The median follow-up time was 17.0 months (IQR 25% 9.8–IQR 75% 27.0). By the time the follow-up period ended, 11/22 patients were still on Lenvatinib treatment. The mean time from baseline ^18^F-FDG-PET/CT to treatment initiation was 1.2 months (range 0–4.0), the first follow-up ^18^F-FDG-PET/CT was performed after a mean of 3.7 months (range 2.0–6.0) and the second follow-up occurred after a mean of 5.6 months (range 3.0–8.0).

#### 2.2.1. Treatment Response According to mPERCIST and RECIST

**Figure 1:**
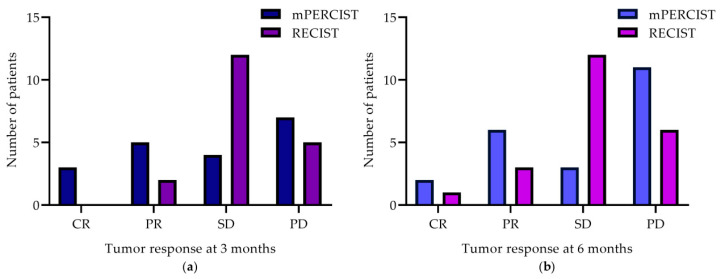
Tumor response evaluation by mPERCIST and RECIST criteria at 3 month (**a**) and 6 month (**b**) follow-up imaging. mPERCIST, modified Positron Emission Tomography Response Criteria In Solid Tumors; RECIST, Response Evaluation Criteria in Solid Tumors; CR, complete response; PR, partial response; SD, stable disease; PD, progressive disease.

After 3.7 ± 1.0 months, data of 19 patients were available for response evaluation. Of those 19 patients, 12/19 patients showed disease control (DC) according to mPERCIST and 14/19 showed DC according to RECIST, whereas 7/19 patients showed progressive disease (PD) according to mPERCIST and 5/19 patients according to RECIST. None of the enrolled patients showed complete response (CR) in morphological evaluation (RECIST), though 3 patients showed CR in a metabolic assessment (see Figure 1a). 

After 5.7 ± 1.4 months, 22 patients were evaluated. Compared to baseline, more patients showed PD (mPERCIST/RECIST: 11/6), but 2 patients indicated CR according to mPERCIST and one patient according to RECIST (see Figure 1b). 

For both evaluation time-points, the majority of patients were rated SD in morphological evaluation (3 months: *n* = 12, 6 months: *n* = 12), while there was more heterogeneity in metabolic treatment response, with most patients presenting with PD. Details of treatment response at 3 and 6 months are presented in Figure 1 and single-patient course of disease are summarized in Table S2 in the supplemental file.

#### 2.2.2. Additional Single PET-Parameters

When evaluating single PET-parameters (mean/maximum standardized uptake value (SUV_mean/max_), Metabolic Tumor Volume (MTV) and Total Lesion Glycolysis (TLG)) additionally to mPERCIST classification according to peak standardized uptake value (SUV_peak_) measurements, all responders (RECIST) showed a decline in all PET-parameters from baseline to the 3 month follow-up and from baseline to the 6 month follow-up, except SUV_mean_ from baseline to the 6 month follow-up. 

**Figure 2:**
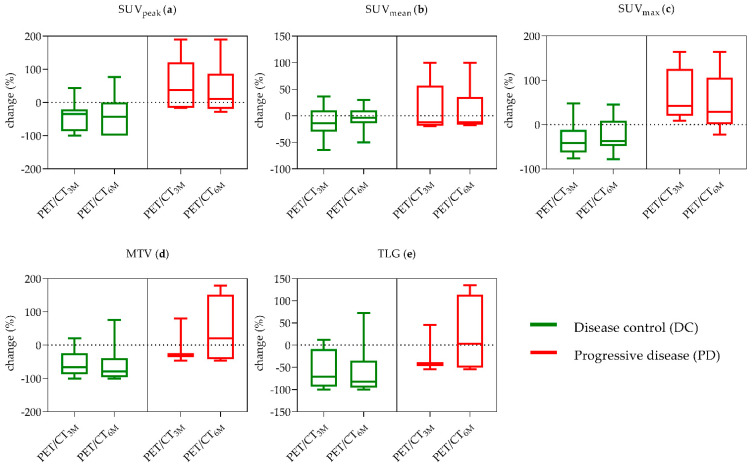
Mean changes of PET-parameters (**a**) SUV_peak_, (**b**) SUV_mean_, (**c**) SUV_max_, (**d**) MTV and (**e**) TLG in patients with DC and PD according to RECIST from baseline to 3 and 6 month follow-up imaging. 3M, 3 months; 6M, 6 months; DC, disease control; PD, progressive disease; SUV, standard uptake value; MTV, metabolic tumor volume; TLG, total lesion glycolysis.

The mean changes of all PET-parameters over 6 months follow-up divided into DC/PD (RECIST) and significant differences between groups are shown in Table 1 and Figure 2. For both time-points, DC patients showed significantly lower SUV_peak_ (3 months: *p* = 0.004, 6 months: *p* = 0.023), SUV_max_ (3 months: *p* = 0.003, 6 months: *p* = 0.008), MTV (3 months: *p* = 0.010, 6 months: *p* = 0.006) and TLG values (3 months: *p* = 0.019, 6 months: *p* = 0.011). One patient was excluded due to not measurable uptake in baseline ^18^F-FDG-PET/CT. The percentage change of Tg-levels from baseline to the 3 month follow up and baseline to the 6 month follow-up ^18^F-FDG-PET/CT showed no significant difference between DC and PD patients (baseline to 3 months: −41% ± 72%, *p* = 0.853; baseline to 6 months: −45% ± 73%, *p* = 0.436).

### 2.3. Outcome Analysis

During the follow-up period, 13/22 patients progressed (according to RECIST). The mean PFS was 12.5 ± 9.2 months. At the end of the study, 12/22 patients had died of thyroid cancer. The mean DSS was 14.2 ± 9.1 months. For detailed PFS and DSS of all patients, see Table S2 in the supplemental information.

**Figure 3:**
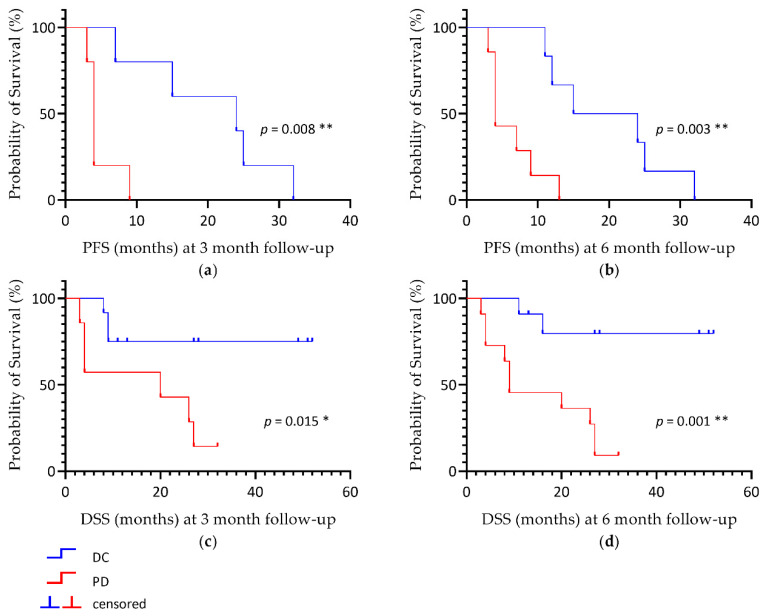
Kaplan-Meier estimate of PFS (**a**,**b**) and DSS (**c**,**d**) according to mPERCIST criteria for 3 and 6 month follow-up imaging. PFS, progression-free survival; DSS, disease-specific survival; DC, disease control; PD, progressive disease; * *p*-value < 0.05; ** *p*-value < 0.01.

PD according to mPERCIST was correlated with worse outcomes and significantly lower PFS and DSS for both time-points. Patients with PD at 3 month follow-up showed a significant lower median PFS (4.0 vs. 24 months, *p* = 0.008) and DSS (20.0 months vs. median not reached for responders, *p* = 0.015) compared to DC (see Figure 3a,c). At the 6 month follow-up, mPERCIST also showed significant differences for both PFS and DSS (PFS 4.0 vs. 15.0 months, *p* = 0.003; DSS 9.0 months vs. the median not reached for responders, *p* = 0.001) between DC and PD (see Figure 3b,d). 

According to the RECIST criteria, PFS did not differ between PD and DC patients at 3 months (15.0 versus 4.0 months, *p* = 0.196) and 6 months (13.0 versus 4.0 months, *p* = 0.114). Patients with PD by RECIST showed a significantly lower median DSS at 3 months (4.0 months versus median not reached, *p* = 0.046) and 6 months (6.5 months versus median not reached, *p* = 0.039; see Table 2).

All other PET-parameters showed no significant association to PFS and DSS for both time-points.

## 3. Discussion

In the present study, we investigated the role of ^18^F-FDG-PET/CT for monitoring functional tumor response in comparison to morphological imaging and its ability to predict PFS and DSS in patients with advanced, radioiodine refractory DTC undergoing Lenvatinib treatment. Our results demonstrate that functional imaging is able to evaluate tumor response in a more differentiated manner than morphological imaging only. In our series, all patients who responded to therapy showed a decline in all PET-parameters except SUV_mean_, whereas a lack of functional tumor response was associated with a worse outcome (PFS and DSS). PET-parameters SUV_peak_, SUV_max_, MTV and TLG at 3 and 6 month follow-ups were significantly higher in patients with disease progression and could serve as additional markers for monitoring early tumor response and outcome. 

The current standard for monitoring treatment response and progression in clinical trials is the change in tumor size assessed by RECIST. To date, the current guidelines do not give specific recommendations for response monitoring in patients with DTC outside clinical trials and response assessment by PERCIST is not mentioned [19]. Since clinical patient management and treatment planning depend on response monitoring through imaging, clinical decisions may vary based on different imaging modalities. 

^18^F-FDG-PET/CT combines functional and morphological imaging. Measurement of glucose metabolism varies less than tumor size measurements and can better distinguish between active tumor and post-therapeutic changes [20,21]. Tumor response evaluation using ^18^F-FDG-PET/CT showed promising results for several cancer entities, such as breast, lung and pancreatic cancer [17,21,22,23]. In addition, many previous studies have shown the important role of ^18^F-FDG-PET/CT in staging and follow-up of patients with advanced, metastatic DTC [24,25,26] and its valuable role in patients with metastatic DTC under treatment with TKIs [27,28]. However, to date the number of studies in patients with DTC undergoing Lenvatinib treatment is limited and no standardized treatment assessment has been proposed so far.

Tumor FDG-uptake can be measured in various ways. Firstly, we used the single-lesion method according to Wahl et al. [29], which was shown to be superior to the five-lesion method [22,30]. Secondly, in accordance to Fendler et al., we used peak standardized uptake value corrected for body weight (SUV_peak_) instead of lean body mass corrected SUV (SUL_peak)_ as proposed by PERCIST 1.0, because the main objective is the percentage change of SUV from baseline to follow-up imaging and should therefore not be a significant confounder [31]. Riedl et al. could also show that response classification was unchanged when SUV_max_ was used instead of SUL_peak_ in patients with metastatic breast cancer [22]. Thus, we measured the SUV_peak_ of the most active lesion, which may differ in consecutive scans. Furthermore, we assessed the PET-parameters SUV_mean_, SUV_max_, MTV and TLG to identify possible other measures since technical methods for quantitative measurement are under continuous improvement [32]. 

Novel cancer therapies, such as TKI treatment, are cytostatic rather than cytotoxic and therefore may not result in a significant decrease of tumor size [33,34]. Additionally, certain metastasis localizations such as bone metastasis do not frequently show morphological changes after therapy [22]. Thus, differentiation of tumor response categorization in CR, PR, SD and PD in these patients by morphological imaging is limited. However, FDG-uptake in tumor cells is known to correlate with disease prognosis [35] and, as precision therapy is evolving, the current monitoring of treatment response does not seem to have been adjusted accordingly. 

Whereas the majority of patients in our study were categorized as SD by RECIST 1.1 on CT at both follow-up times, our data show that patients were categorized in a more differentiated manner by mPERCIST using ^18^F-FDG-PET. This finding is in line with the study of Riedl et al., who reported that patients with metastatic breast cancer with SD and PD by RECIST were frequently classified worse by PERCIST [22].

Based on these differences in categorization of patients, Kaplan-Meier analysis demonstrated significant distinction between DC and PD. Tumor response by mPERCIST was significantly correlated with PFS and DSS, whereas tumor response by morphological imaging showed no significant correlation. In our study, responses determined by using the RECIST 1.1 criteria at 3 and 6 months was found to be statistically significant for DSS, but the association was lower compared to the mPERCIST response, indicating that mPERCIST detects progression more precisely than RECIST. All other PET-parameters (SUV_mean/max_, MTV and TLG) showed no significant association with PFS and DSS. These findings are consistent with the results reported by Riedl et al. [22] and could subsequently lead to earlier change of therapy in patients considered SD by RECIST but do not show therapy response by mPERCIST. The association of metabolic response with survival benefit has already been shown in several other solid tumors such as breast cancer [36]. 

In this study, a decline of nearly all PET-parameters was found in patients with DC. Studies on the role of the PET-parameter SUV_max_ in DTC patients are limited. In line with our data, Carr et al. showed that patients with metastatic DTC or medullary thyroid carcinoma treated with Sunitinib showing DC had a significant decline in average and mean percentage change SUV_max_ compared to patients with progressive disease [27]. A significant decline in SUV_max_ could also be shown in patients with radioiodine refractory DTC treated with Apatinib, whereas in DTC patients treated with Vandetanib, no correlation between SUV_max_ and DC could be found [37]. To date, SUV_max_ is the most commonly used semiquantitative PET-parameter due to its simple application but there are more and more studies suggesting the use of SUV_peak_ alternatively [38]. Since SUV_peak_ is measured in a larger VOI than the single-pixel SUV_max_, it appears to be more robust to image noise.

The volume-related PET-parameters MTV and TLG could also show promising results in metastatic DTC patients. The study by Manohar et al. showed that these parameters can be used for dynamic risk stratification regarding PFS [39]. TLG appeared promising in some cancers such as colorectal cancer and brain tumors, but not in others such as sarcomas [40,41,42,43]. Data from Lee et al. showed that in patients with pancreatic cancer, TLG was an independent prognostic factor for predicting recurrence-free and overall survival, whereas Benz et al. reported that TLG was less accurate in predicting tumor response in sarcomas compared to SUV_mean_ and SUV_max_ [23,40]. The same was shown for MTV, which was found to be useful for treatment response assessment in non-small cell lung cancer (NSCLC) and pancreatic cancer [23,44]. Furthermore, it was demonstrated to be an independent prognostic factor for DSS in patients with cervical cancer treated by radical surgery and could independently predict survival in patients with locally advanced squamous cell cervical carcinoma [45,46]. Indeed, in our analysis, volume related PET-parameters (MTV and TLG) were found to be useful tools to distinguish DC from PD but failed to provide prognostic value in terms of PFS and DSS. SUV_mean_ showed the weakest correlation with tumor response in our study. In contrast, Werner et al. identified a SUV_mean_ of less than 4.0 before treatment in medullary thyroid carcinoma as a predictor of longer PFS [20]. Consequently, all PET-parameters except for SUV_mean_ seem to be a useful tool and may be evaluated alongside SUV_peak_ as suggested in PERCIST 1.1 criteria [29]. 

The percentage change of Tg-levels from baseline to 3 month- and baseline to 6 month follow-up ^18^F-FDG-PET/CT showed no significant difference between DC and PD patients. One possible explanation for the missing correlation between Tg and ^18^F-FDG-PET/CT might be the different de-differentiation level of the thyroid cancer patients treated with Lenvatinib.

To our knowledge, this is the first study attempting to address the impact of using different imaging modalities and therefore different evaluation criteria for treatment response in patients with advanced, radioiodine refractory DTC undergoing Lenvatinib treatment. However, the role of ^18^F-FDG-PET/CT and the selection of the optimal PET-parameters for monitoring of functional tumor response in patients with advanced, radioiodine refractory DTC undergoing Lenvatinib treatment will have to be verified in prospective trials in larger patient cohorts.

Our study has several limitations. Due to the retrospective design of the study, the span between baseline imaging and initiation of treatment and the intervals of follow-up imaging varied between patients and follow-up imaging at 3 months was not available in all patients. Furthermore, due to the small cohort, the rarity of the disease and heterogeneity in patient cohort (histology, stage of disease), statistical power of the analysis is limited. 

## 4. Materials and Methods 

### 4.1. Study Population

For this retrospective study, we selected patients with advanced, radioiodine-refractory PTC, FTC and PDTC undergoing Lenvatinib treatment at the department of endocrinology (University Hospital, LMU Munich, Munich, Germany) between May 2015 and August 2019. Patients were eligible for inclusion in this study when the time span between baseline ^18^F-FDG-PET/CT and initiation of treatment was less than 4 months and follow-up imaging was performed after 3 ± 3 months and/or 6 ± 3 months at the Department of Nuclear Medicine (University Hospital, LMU Munich, Munich, Germany). 

All patients had extended disease, were radioiodine-refractory and had at least one FDG-positive lesion, except for one patient at baseline PET scan. Lenvatinib treatment was initiated based on a multidisciplinary tumor board decision. 

### 4.2. Ethics Statement

The study was approved by the local ethics committee (Ethics committee of the Medical Faculty,

University Hospital, LMU Munich, Munich, Germany, IRB #20-736, 21.09.2020) and has been conducted in accordance with the ethical standards of the Declaration of Helsinki and national and international guidelines. The requirement to obtain informed consent was waived due to the retrospective design of this study.

### 4.3. Imaging Techniques

Patients fasted at least 6 h (demanded glucose level <160 mg/dL). Prior to the injection of approximately 250 MBq ^18^F-FDG whole-body, ^18^F-FDG-PET/CT images were acquired using a Biograph 64 TruePoint w/TrueV and Biograph mCT Flow 20-4R PET/CT scanner (Siemens, Healthcare GmbH, Erlangen, Germany) and were initiated approximately 60 min after intravenous tracer administration. After intravenous injection of a contrast agent (Ultravist 300, Bayer Vital GmbH, Leverkusen, Germany or Imeron 350, 2.5 mL/s, Bracco Imaging Deutschland GmbH, Konstanz, Germany) diagnostic CT scans of the neck, thorax, abdomen and pelvis (100–190 mAs; 120 kV) were acquired. 

To depict the venous phase, initiation of CT acquisition was delayed 90 s after injection of the contrast agent.

### 4.4. Response Evaluation

Complete response (CR), partial response (PR) and stable disease (SD) were considered as disease control (DC), remaining patients were categorized as progressive disease (PD) using both RECIST 1.1 in CT and modified PERCIST criteria (mPERCIST) in ^18^F-FDG-PET of combined ^18^F-FDG-PET/CT.

#### 4.4.1. RECIST 1.1

The implementation of the evaluation criteria RECIST 1.1 is based on the original publication of Eisenhauer et al. [15]. RECIST 1.1 was performed by an experienced radiologist using mint Lesion version 3.7 software (Mint Medical GmbH, Heidelberg, Germany) without knowledge of the results of the PET studies. A maximum of 3 to 5 target lesions were measured (max. 2 target lesions per organ) and at least 2 non-target lesions at baseline, 3 month and 6 month follow-up CT-scans. Time-point responses were then evaluated automatically. A relative increase of 20% and an absolute increase of at least 5 mm in target sum were considered as PD, while a decrease of 30% in target sum was considered PR.

#### 4.4.2. mPERCIST and Other PET-Parameters

Semi-automated measurements of PET-parameters in ^18^F-FDG-PET of the entire tumor burden in all patients were performed by an experienced nuclear medicine physician using image fusion software (Hybrid Viewer 2.6, Hermes Medical Solutions, Stockholm, Sweden) [29]. First the background was measured in the healthy liver to ensure the technical comparability of PET studies and to determine if the target tumor lesion shows sufficiently high glucose uptake with a minimum threshold for measurability defined as 1.5 × (mean value of normal liver) + 2 × (standard deviation of liver) or greater at baseline. For measurements of the background activity a fixed 3-cm diameter spherical volume of interest (VOI) was placed in the right side of the liver. Furthermore, a new lesion or unequivocal progression in the follow-up PET scan was rated as progressive disease changes, even if the target lesion at baseline did not show the minimum glucose uptake.

To assess treatment response by mPERCIST change in peak standardized uptake value (SUV_peak_) was measured in the tumor region with the highest radiotracer uptake, which describes the average SUV computed in a fixed 1-mL sphere recommended by PERCIST instead of the widely used single-pixel maximum standardized uptake value (SUV_max_) to avoid noise errors [18,29]. In a slight modification to the PERCIST 1.0 criteria, the quantitative PET-parameter was adjusted to body weight (SUV_peak_ in g/mL) rather than the body surface area (SUL_peak_), as previously described by Fendler et al. for the single most active lesion in the patient at baseline, 3 month and 6 month follow-up [31]. A 1 mL spherical VOI was placed at the focus of the single active lesion and the highest SUV_peak_ value was computed automatically. The single most active lesion presents the target lesion, from which it is assumed to correspond to the worst behaving portion of the tumor. The percentage changes in SUV_peak_ from the reference baseline scan to 3 month and 6 month follow-up scans were assessed in all patients. In mPERCIST, decrease greater than or equal to 30% in SUV_peak_ was considered as PR and increase greater than 30% as PD (see Table 3). 

All further PET-parameters were derived through segmentation of all tumor lesions: mean/maximum standardized uptake value (SUV_mean/max_), Metabolic Tumor Volume (MTV) and Total Lesion Glycolysis (TLG). MTV was defined by volume delineation and TLG was calculated as (MTV x SUV_mean_) [23]. All areas with physiological, non-tumoral ^18^F-FDG-uptake were excluded. Response was determined separately in all PET-parameters using the same criteria for percentage change except for TLG, which had to show an increase of greater than 75% according to Wahl et al. to be assessed as PD (see Table 3) [29].

### 4.5. Outcome Analysis

PFS was defined as the time between treatment start and disease progression according to RECIST 1.1. Disease-specific survival (DSS) was calculated from the time of baseline ^18^F-FDG-PET/CT until time of death. The observation period ended on 26th of May 2020.

### 4.6. Statistical Analysis

Ordinal and continuous variables are presented as median (interquartile range, IQR) or mean ± standard deviation (SD). Change in % was calculated using the following formula: ((value of follow-up PET)/(value of baseline PET) − 1) × 100. The Mann-Whitney u test was used to compare mean percentage changes between DC and PD patients. Survival analysis using Kaplan-Meier analysis was performed for PFS and DSS according to the mPERCIST and RECIST 1.1 criteria for 3 month and 6 month follow-ups. Quantitative survival data are given as median in months. Log rank test was used to compare survival rates between subgroups. *p*-values ≤ 0.05 were considered to indicate statistical significance. All analyses were performed using SPSS computer software (SPSS Statistics 25, IBM).

## 5. Conclusions

In conclusion, our study suggests that in patients with advanced radioiodine refractory DTC undergoing Lenvatinib treatment, tumor response evaluation by ^18^F-FDG-PET/CT outperforms morphological response evaluation using CT and furthermore appears to be stronger correlated with outcome analysis. Monitoring tumor response with ^18^F-FDG-PET/CT in these patients has the potential to improve treatment individualization and avoid ineffective therapies by selecting patients who will most likely benefit from Lenvatinib treatment. Therefore, tumor response assessed by ^18^F-FDG-PET/CT is a highly promising modality in order to increase diagnostic accuracy in these patients and should be further investigated.

## Figures and Tables

**Table 1:** Mean changes and SD (%) of PET-parameters in patients with DC and PD according to RECIST from baseline to 3 and 6 month follow-up imaging.

Mean Change ±SD (%)	SUV_peak_	SUV_mean_	SUV_max_	MTV	TLG
Baseline to 3 Month Follow-Up					
DC (n = 13/18)	−43% ± 45%	−12% ± 28%	−33% ± 35%	−56% ± 38%	−56% ± 42%
PD (n = 5/19)	49% ± 84%	12% ± 51%	66% ± 62%	439% ± 718%	797% ± 1489%
*p*-value	0.004 **	0.336	0.003 **	0.010 *	0.019 *
Baseline to 6 Month Follow-Up					
DC (n = 15/21)	−42% ± 57%	−2% ± 21%	−24% ± 35%	−23% ± 152%	−10% ± 189%
PD (n = 6/22)	36% ± 81%	9% ± 46%	49% ± 68%	394% ± 652%	687% ± 1358%
*p*-value	0.023 *	0.677	0.008 **	0.006 **	0.011 *

DC, disease control; PD, progressive disease; SD, standard deviation; SUV, standard uptake value; MTV, metabolic tumor volume; TLG, total lesion glycolysis; * *p*-value < 0.05; ** *p*-value < 0.01.

**Table 2:** Association of PD and DC according to mPERCIST and RECIST to PFS and DSS at the 3 month and 6 month follow-ups.

*p*-Value	mPERCIST		RECIST	
	3 Months	6 Months	3 Months	6 Months
PFS	0.008 **	0.003 **	0.196	0.114
DSS	0.015 *	0.001 **	0.046 *	0.039 *

mPERCIST, modified Positron Emission Tomography Response Criteria In Solid Tumors; RECIST, Response Evaluation Criteria in Solid Tumors; DC, disease control; PD, progressive disease; * *p*-value < 0.05; ** *p*-value < 0.01.

**Table 3:** mPERCIST criteria from Wahl [29], modified by Fendler et al. [31].

Response Criteria	
Complete response (CR)	Normalization of all lesions to SUVpeak less than mean liver SUV and indistinguishable from surrounding background
Partial response (PR)	>30% decrease in SUVpeak; minimum 0.8 unit decrease in SUVpeak
Stable disease (SD)	Does not meet other criteria
Progressive disease (PD)	>30% increase in SUVpeak; minimum 0.8 unit increase in SUVpeak>75% increase in TLG

Outcome determination is measured on the single most active lesion on each scan (not necessarily the same lesion). Standardized uptake value (SUV).

## Data Availability

The data presented in this study are available upon reasonable request from the corresponding author.

## Supplementary Materials

The following are available online at https://www.mdpi.com/2072-6694/13/2/317/s1, Table S1: Patient Characteristics, Table S2: Single-Patient Course of Disease.
