# Supplementary material for: 18F-FDG-PET/CT in Patients with Advanced, Radioiodine Refractory Thyroid Cancer Treated with Lenvatinib
Source: Cancers (Basel). 2021 Jan 16;13(2):317. doi: 10.3390/cancers13020317 (PMC7830971; doi:10.3390/cancers13020317)
Supplement: Supplementary file 1 [file cancers-13-00317-s001.pdf]

cancers

MDPI

Supplementary Materials: ^{18}F-FDG-PET/CT in Patients with Advanced, Radioiodine Refractory Thyroid Cancer Treated with Lenvatinib

Freba Ahmaddy, Caroline Burgard, Leonie Beyer, Viktoria Florentine Koehler, Peter Bartenstein, Matthias P. Fabritius, Thomas Geyer, Vera Wenter, Harun Ilhan, Christine Spitzweg and Andrei Todica

Table S1. Patient Characteristics. PP, primary presentation; F, female; M, male; FTC, follicular thyroid cancer, PTC, papillary thyroid cancer; PDTC, poorly differentiated thyroid cancer; PUL, pulmonary metastases; M, distant metastasis; NOD, lymph node metastases; OSS, bone metastases; HEP, liver metastases; LR, local recurrence; CER, cerebral metastases, PLEU, pleural metastases, KID, kidney metastases; ST, subcutaneous metastases; OP, surgery, RAD, radiotherapy; CT, chemotherapy; TKI, tyrosin kinase inhibitor therapy other than Lenvatinib; REDIFF; redifferentiation therapy with Dabrafenib; DC, disease control; PD, progressive disease.; PFS, progression-free survival; DSS, disease-specific survival.

Patient No.	Gender	Age at PP (Years)	Age at Lenvatinib Start (Years)	Histology	Stage	Sites of Disease at PP	Sites of Disease at Lenvatinib Start	Prior Therapies
1	F	49	55	FTC	II	M0	PUL	OP, RAD
2	M	31	44	PDTC	IV	M0	PUL, HEP, OSS	RAD
3	M	73	75	PDTC	IV	M0	PUL, NOD	OP, RAD
4	M	51	53	PTC	III	M0	LR, PUL, NOD	OP, RAD, REDIFF
5	F	49	83	FTC	IV	M0	LR, PUL, OSS, KIDN	OP, RAD
6	F	65	65	PDTC	IV	M0	PUL	RAD
7	F	69	80	FTC	IV	M0	PUL, PLEU, OSS	OP, RAD
8	F	43	45	PDTC	III	M0	LR	OP, RAD, TKI
9	M	51	52	PDTC	IV	M0	LR	OP, RAD, TKI
10	F	50	56	FTC	III	PUL	PUL, NOD	OP, RAD
11	F	61	62	PTC	IV	PUL	PUL, NOD	TKI

12	F	43	53	FTC	III	OSS	OSS, PUL, NOD	RAD, TKI
13	M	33	46	FTC	II	PUL	PUL, NOD, CER, HEP, OSS	OP, RAD, TKI
14	F	33	35	FTC	III	PUL	PUL, LR, NOD	OP, RAD
15	M	56	58	PTC	III	ST	ST, PUL, NOD	OP, TKI
16	M	77	77	PDTC	IV	PUL	PUL, PLEU	-
17	F	71	84	FTC	IV	Mx	NOD	OP
18	M	64	67	FTC	III	Mx	PUL, NOD	-
19	F	49	62	FTC	III	Mx	PUL, NOD	-
20	M	57	76	FTC	II	Mx	PUL	RAD, TKI
21	M	42	57	FTC	IV	Mx	LR, PUL	OP
22	M	51	52	PDTC	III	Mx	PUL, NOD	OP, RAD, CT, TKI

Table S2. Single-Patient Course of Disease. 3M, 3 months; 6M, 6 months; RECIST, Response Evaluation Criteria in Solid Tumors; mPERCIST, modified Positron Emission Tomography Response Criteria In Solid Tumors; PFS, progression-free survival; DSS, disease-specific survival; DC, disease control; PD, progressive disease; †, dead; --, alive.

Patient No.	3M RECIST	3M PERCIST	6M RECIST	6M PERCIST	PFS (Months)	DSS (Months)	Death
1	DC	DC	DC	DC	--	--	--
2	DC	DC	DC	DC	--	--	--
3	DC	DC	DC	PD	--	8	†
4	--	--	DC	PD	13	27	†
5	DC	PD	DC	PD	--	20	†
6	DC	DC	DC	PD	--	9	†

7	DC	DC	PD	PD	7	9	†
8	--	--	DC	DC	12	16	†
9	DC	DC	DC	DC	15	--	--
10	--	--	DC	DC	11	16	†
11	DC	PD	DC	PD	9	26	†
12	DC	PD	DC	PD	--	--	--
13	PD	PD	PD	PD	4	27	†
14	DC	DC	DC	DC	--	--	--
15	DC	DC	DC	DC	32	--	--
16	PD	PD	PD	PD	4	4	†
17	DC	DC	DC	DC	--	--	--
18	DC	DC	DC	DC	--	--	--
19	DC	DC	DC	DC	24	--	--
20	PD	DC	PD	DC	25	--	--
21	PD	PD	PD	PD	4	4	†
22	PD	PD	PD	PD	3	4	†

© 2021 by the authors. Licensee MDPI, Basel, Switzerland. This article is an open access article distributed under the terms and conditions of the Creative Commons Attribution (CC BY) license (http://creativecommons.org/licenses/by/4.0/).
